# (2*E*)-2-(4-Hy­droxy-3-meth­oxy­benzyl­idene)hydrazinecarboxamide

**DOI:** 10.1107/S1600536812025299

**Published:** 2012-06-13

**Authors:** M. Nawaz Tahir, Akbar Ali, M. Naveed Umar, Ishtiaq Hussain, Hazoor Ahmad Shad

**Affiliations:** a Department of Physics, University of Sargodha, Sargodha, Pakistan; b Department of Chemistry, University of Malakand, Pakistan; c Department of Chemistry, University of Sargodha, Pakistan; d Department of Chemistry, Government Post Graduate College, Gojra, Punjab, Pakistan

## Abstract

In the title compound, C_9_H_11_N_3_O_3_, two mol­ecules are present in the asymmetric unit in which the 4-hy­droxy-3-meth­oxy­benzaldehyde and hydrazinecarboxamide units are almost planar [with r.m.s. deviations 0.0212 and 0.0066 Å, respectively, in one mol­ecule and 0.0346 and 0.0095 Å, respectively, in the other] and are oriented at dihedral angles of 9.7 (3) and 16.6 (3)°. In both mol­ecules, two *S*(5) ring motifs are present due to N—H⋯N and O—H⋯O hydrogen bonds. In the crystal, the mol­ecules are dimerized with each other due to pairs of N—H⋯O hydrogen bonds, forming an *R*_2_^2^(8) ring motif. O—H⋯O hydrogen bonds lead to the formation of a three-dimensional network.

## Related literature

For a related structure, see: Tahir *et al.* (2012 ▶). For graph–set notation, see: Bernstein *et al.* (1995 ▶).
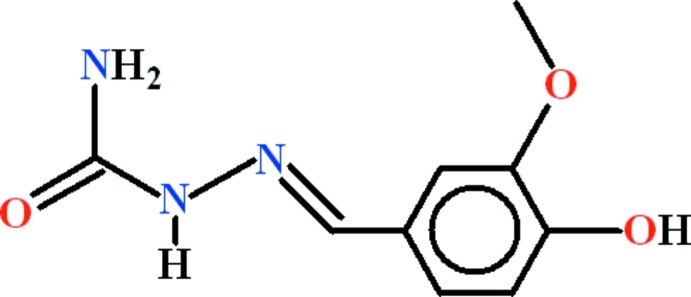


## Experimental

### 

#### Crystal data

C_9_H_11_N_3_O_3_
*M* *_r_* = 209.21
Orthorhombic, 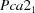

*a* = 13.9945 (14) Å
*b* = 5.0440 (4) Å
*c* = 27.286 (2) Å
*V* = 1926.0 (3) Å^3^
*Z* = 8
Mo *K*α radiation
μ = 0.11 mm^−1^
*T* = 296 K
0.30 × 0.16 × 0.14 mm

#### Data collection

Bruker Kappa APEXII CCD diffractometer
Absorption correction: multi-scan (*SADABS*; Bruker, 2005 ▶) *T*_min_ = 0.957, *T*_max_ = 0.966
8297 measured reflections
1931 independent reflections
1046 reflections with *I* > 2σ(*I*)
*R*_int_ = 0.081

#### Refinement

*R*[*F* ^2^ > 2σ(*F* ^2^)] = 0.056
*wR*(*F* ^2^) = 0.113
*S* = 0.98
1931 reflections
275 parameters
H-atom parameters constrained
Δρ_max_ = 0.22 e Å^−3^
Δρ_min_ = −0.25 e Å^−3^

### 

Data collection: *APEX2* (Bruker, 2007 ▶); cell refinement: *SAINT* (Bruker, 2007 ▶); data reduction: *SAINT*; program(s) used to solve structure: *SHELXS97* (Sheldrick, 2008 ▶); program(s) used to refine structure: *SHELXL97* (Sheldrick, 2008 ▶); molecular graphics: *ORTEP-3 for Windows* (Farrugia, 1997 ▶) and *PLATON* (Spek, 2009 ▶); software used to prepare material for publication: *WinGX* (Farrugia, 1999 ▶) and *PLATON*).

## Supplementary Material

Crystal structure: contains datablock(s) global, I. DOI: 10.1107/S1600536812025299/bq2364sup1.cif

Structure factors: contains datablock(s) I. DOI: 10.1107/S1600536812025299/bq2364Isup2.hkl

Supplementary material file. DOI: 10.1107/S1600536812025299/bq2364Isup3.cml

Additional supplementary materials:  crystallographic information; 3D view; checkCIF report

## Figures and Tables

**Table 1:** Hydrogen-bond geometry (Å, °)

*D*—H⋯*A*	*D*—H	H⋯*A*	*D*⋯*A*	*D*—H⋯*A*
O2—H2*A*⋯O1	0.82	2.17	2.627 (6)	115
O2—H2*A*⋯O5^i^	0.82	2.34	3.108 (7)	156
N2—H2*B*⋯O6^ii^	0.86	2.11	2.923 (7)	158
N3—H3*A*⋯O6^iii^	0.86	2.16	2.987 (7)	162
N3—H3*B*⋯N1	0.86	2.31	2.674 (8)	106
O5—H5*A*⋯O4	0.82	2.18	2.632 (6)	115
N5—H5*B*⋯O3^iv^	0.86	2.08	2.909 (7)	161
N6—H6*A*⋯O3^v^	0.86	2.13	2.965 (7)	164
N6—H6*B*⋯N4	0.86	2.32	2.677 (7)	105

Symmetry codes: (i) 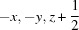; (ii) 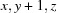; (iii) 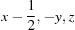; (iv) 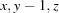; (v) 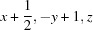.

**Fig. 1.**
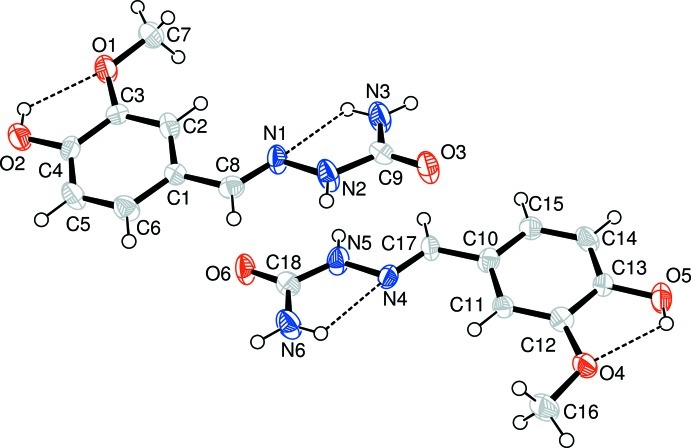
View of the title compound with the atom numbering scheme. The thermal ellipsoids are drawn at the 50% probability level.

**Fig. 2.**
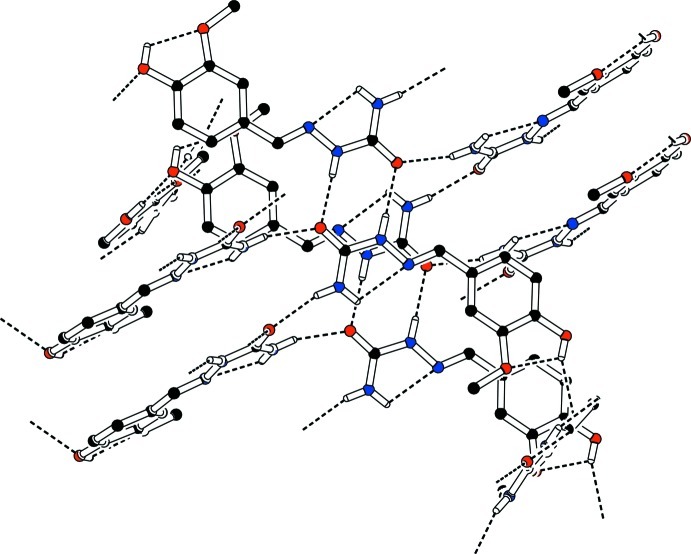
The partial packing (PLATON; Spek, 2009) which shows that molecules form ring motifs in three-dimensional polymeric network.

## supplementary crystallographic information

### Comment

The title compound (I), (Fig. 1) has been synthesized as a derivative. Recently we have reported the crystal structure of (2*E*)-2-(3,4-dimethoxybenzylidene)hydrazinecarboxamide (Tahir *et al.*, 2012) which is related to the title compound.

In (I), two molecules are present in the asymmetric unit, which differ slightly from each other geometrically. In one molecule, the parts of 4-hydroxy-3-methoxybenzaldehyde and hydrazinecarboxamide A (C1—C8/O1/O2) and B (N1/N2/C9/N3/O3), are almost planar with r.m.s. deviations of 0.0212 Å and 0.0066 Å, respectively. The dihedral angle between A/B is 16.57 (26)°. In the second molecule, the similar groups C (C10—C17/O4/O5) and D (N4/N5/C18/N6/O6) are also planar with r.m.s. deviations of 0.0346 Å and 0.0095 Å, respectively, and the dihedral angle between C/D is 9.74 (28)°. In both molecules two *S*(5) ring motifs (Bernstein *et al.*, 1995) are present due to H–bonding of N—H···N and O—H···O types (Table 1, Fig. 1). The molecules are dimerized with each other due to N—H···O type of H-bondings and form *R*_2_^2^(8) ring motifs (Table 1, Fig. 2). The molecules are stabilized in the form of three-dimensional polymeric network.

### Experimental

Equimolar quantities of 4-hydroxy-3-methoxybenzaldehyde and hydrazinecarboxamide were refluxed in methanol along with few drops of acetic acid as catalyst for 45 min resulting in light orange solution. The solution was kept at room temperature which afforded light orange needles after few days.

### Refinement

In the absence of anomalous scattering all Friedel pairs were merged. The H-atoms were positioned geometrically (C–H = 0.93—0.96 Å, N—H = 0.86 Å, O—H = 0.82 Å) and refined as riding with *U*_iso_(H) = *xU*_eq_(C, N, O), where *x* = 1.5 for hydroxy and methyl and *x* = 1.2 for other H-atoms.

### Crystal data

**Table:**

C_9_H_11_N_3_O_3_	*F*(000) = 880
*M**_r_* = 209.21	*D*_x_ = 1.443 Mg m^−^^3^
Orthorhombic, *P**c**a*2_1_	Mo *K*α radiation, λ = 0.71073 Å
Hall symbol: P 2c -2ac	Cell parameters from 2704 reflections
*a* = 13.9945 (14) Å	θ = 1.8–26.0°
*b* = 5.0440 (4) Å	µ = 0.11 mm^−^^1^
*c* = 27.286 (2) Å	*T* = 296 K
*V* = 1926.0 (3) Å^3^	Needle, colorless
*Z* = 8	0.30 × 0.16 × 0.14 mm

### Data collection

**Table:**

Bruker Kappa APEXII CCD diffractometer	1931 independent reflections
Radiation source: fine-focus sealed tube	1046 reflections with *I* > 2σ(*I*)
Graphite monochromator	*R*_int_ = 0.081
Detector resolution: 8.00 pixels mm^-1^	θ_max_ = 26.0°, θ_min_ = 2.9°
ω scans	*h* = −17→17
Absorption correction: multi-scan (*SADABS*; Bruker, 2005)	*k* = −3→6
*T*_min_ = 0.957, *T*_max_ = 0.966	*l* = −33→33
8297 measured reflections	

### Refinement

**Table:**

Refinement on *F*^2^	Primary atom site location: structure-invariant direct methods
Least-squares matrix: full	Secondary atom site location: difference Fourier map
*R*[*F*^2^ > 2σ(*F*^2^)] = 0.056	Hydrogen site location: inferred from neighbouring sites
*wR*(*F*^2^) = 0.113	H-atom parameters constrained
*S* = 0.98	*w* = 1/[σ^2^(*F*_o_^2^) + (0.039*P*)^2^] where *P* = (*F*_o_^2^ + 2*F*_c_^2^)/3
1931 reflections	(Δ/σ)_max_ < 0.001
275 parameters	Δρ_max_ = 0.22 e Å^−^^3^
0 restraints	Δρ_min_ = −0.25 e Å^−^^3^

### Special details

**Table:**

Geometry. Bond distances, angles *etc*. have been calculated using the rounded fractional coordinates. All su's are estimated from the variances of the (full) variance-covariance matrix. The cell e.s.d.'s are taken into account in the estimation of distances, angles and torsion angles
Refinement. Refinement of *F*^2^ against ALL reflections. The weighted *R*-factor *wR* and goodness of fit *S* are based on *F*^2^, conventional *R*-factors *R* are based on *F*, with *F* set to zero for negative *F*^2^. The threshold expression of *F*^2^ > σ(*F*^2^) is used only for calculating *R*-factors(gt) *etc*. and is not relevant to the choice of reflections for refinement. *R*-factors based on *F*^2^ are statistically about twice as large as those based on *F*, and *R*- factors based on ALL data will be even larger.

### Fractional atomic coordinates and isotropic or equivalent isotropic displacement parameters (Å^2^)

**Table:**

	*x*	*y*	*z*	*U*_iso_*/*U*_eq_	
O1	0.1256 (3)	−0.4282 (10)	0.51673 (17)	0.051 (2)	
O2	0.3120 (3)	−0.4570 (10)	0.52664 (17)	0.0437 (19)	
O3	−0.0668 (3)	0.7125 (10)	0.3095 (2)	0.0423 (16)	
N1	0.0730 (4)	0.3033 (11)	0.38554 (19)	0.035 (2)	
N2	0.0463 (3)	0.5085 (12)	0.3552 (2)	0.041 (2)	
N3	−0.1032 (4)	0.3268 (12)	0.3479 (2)	0.048 (2)	
C1	0.1988 (4)	0.0965 (14)	0.4299 (2)	0.031 (3)	
C2	0.1392 (5)	−0.0745 (13)	0.4553 (3)	0.035 (3)	
C3	0.1765 (4)	−0.2580 (13)	0.4879 (2)	0.031 (3)	
C4	0.2739 (5)	−0.2735 (12)	0.4950 (2)	0.031 (2)	
C5	0.3343 (4)	−0.1063 (13)	0.4703 (2)	0.039 (3)	
C6	0.2965 (4)	0.0792 (13)	0.4384 (2)	0.037 (3)	
C7	0.0252 (4)	−0.4398 (15)	0.5100 (3)	0.049 (3)	
C8	0.1602 (5)	0.2916 (13)	0.3966 (2)	0.032 (2)	
C9	−0.0445 (4)	0.5255 (14)	0.3362 (2)	0.031 (2)	
O4	−0.0199 (3)	0.9504 (9)	0.10503 (17)	0.0413 (16)	
O5	−0.2032 (3)	0.8811 (10)	0.08760 (18)	0.049 (2)	
O6	0.2021 (3)	−0.1888 (9)	0.31097 (17)	0.0430 (17)	
N4	0.0595 (3)	0.2198 (10)	0.23612 (18)	0.0307 (17)	
N5	0.0884 (4)	0.0189 (11)	0.2668 (2)	0.040 (2)	
N6	0.2384 (4)	0.1926 (10)	0.2705 (2)	0.044 (2)	
C10	−0.0733 (4)	0.4019 (13)	0.1914 (2)	0.031 (2)	
C11	−0.0188 (4)	0.5961 (13)	0.1670 (2)	0.029 (2)	
C12	−0.0626 (5)	0.7582 (13)	0.1326 (2)	0.030 (2)	
C13	−0.1603 (4)	0.7257 (13)	0.1226 (2)	0.032 (3)	
C14	−0.2137 (5)	0.5398 (14)	0.1471 (3)	0.038 (3)	
C15	−0.1697 (4)	0.3796 (13)	0.1809 (2)	0.035 (2)	
C16	0.0808 (4)	0.9841 (14)	0.1099 (3)	0.045 (3)	
C17	−0.0297 (4)	0.2151 (12)	0.2254 (2)	0.030 (2)	
C18	0.1785 (5)	0.0009 (15)	0.2845 (2)	0.033 (2)	
H2	0.07352	−0.06591	0.45048	0.0424*	
H2A	0.26884	−0.54591	0.53860	0.0649*	
H2B	0.08747	0.62892	0.34795	0.0490*	
H3A	−0.16059	0.32439	0.33656	0.0569*	
H3B	−0.08372	0.20124	0.36668	0.0569*	
H5	0.40001	−0.11747	0.47486	0.0464*	
H6	0.33740	0.19515	0.42224	0.0444*	
H7A	0.01136	−0.49086	0.47685	0.0593*	
H7B	−0.00207	−0.26864	0.51642	0.0593*	
H7C	−0.00159	−0.56760	0.53213	0.0593*	
H8	0.20166	0.41433	0.38265	0.0377*	
H5A	−0.16623	0.99847	0.07909	0.0729*	
H5B	0.04750	−0.10020	0.27510	0.0479*	
H6A	0.29709	0.18908	0.27988	0.0534*	
H6B	0.21835	0.31947	0.25217	0.0534*	
H11	0.04595	0.61546	0.17385	0.0351*	
H14	−0.27877	0.52269	0.14088	0.0458*	
H15	−0.20577	0.25249	0.19730	0.0421*	
H16A	0.11246	0.81983	0.10266	0.0675*	
H16B	0.09561	1.03701	0.14284	0.0675*	
H16C	0.10226	1.11826	0.08749	0.0675*	
H17	−0.06819	0.08711	0.23996	0.0368*	

### Atomic displacement parameters (Å^2^)

**Table:**

	*U*^11^	*U*^22^	*U*^33^	*U*^12^	*U*^13^	*U*^23^
O1	0.028 (3)	0.070 (4)	0.055 (4)	−0.003 (3)	−0.004 (3)	0.032 (3)
O2	0.030 (3)	0.052 (4)	0.049 (3)	0.005 (2)	−0.003 (2)	0.013 (3)
O3	0.028 (2)	0.039 (3)	0.060 (3)	0.000 (2)	−0.011 (2)	0.016 (3)
N1	0.031 (3)	0.031 (4)	0.042 (4)	0.001 (3)	−0.010 (3)	0.012 (3)
N2	0.024 (3)	0.036 (4)	0.062 (4)	−0.005 (3)	−0.013 (3)	0.013 (3)
N3	0.026 (3)	0.055 (5)	0.062 (4)	−0.003 (3)	−0.010 (3)	0.020 (4)
C1	0.025 (4)	0.038 (5)	0.030 (4)	0.000 (3)	0.001 (3)	0.005 (3)
C2	0.029 (4)	0.037 (5)	0.040 (4)	0.007 (4)	−0.007 (3)	0.001 (4)
C3	0.029 (4)	0.039 (5)	0.026 (4)	0.000 (3)	0.003 (3)	0.003 (3)
C4	0.036 (4)	0.033 (4)	0.023 (4)	0.010 (4)	−0.005 (3)	−0.002 (3)
C5	0.024 (4)	0.039 (5)	0.054 (5)	0.001 (3)	−0.004 (3)	0.007 (4)
C6	0.029 (4)	0.033 (5)	0.049 (5)	−0.002 (3)	0.003 (3)	0.007 (4)
C7	0.027 (4)	0.074 (6)	0.047 (5)	−0.009 (4)	−0.003 (4)	0.006 (4)
C8	0.033 (4)	0.032 (4)	0.030 (4)	−0.005 (4)	0.003 (3)	0.000 (3)
C9	0.023 (4)	0.032 (4)	0.038 (4)	0.001 (3)	0.002 (3)	−0.005 (4)
O4	0.026 (2)	0.045 (3)	0.053 (3)	0.002 (2)	0.004 (2)	0.018 (3)
O5	0.036 (3)	0.056 (4)	0.054 (4)	0.008 (3)	−0.008 (3)	0.023 (3)
O6	0.037 (3)	0.037 (3)	0.055 (3)	0.006 (2)	−0.013 (3)	0.016 (3)
N4	0.033 (3)	0.032 (3)	0.027 (3)	0.001 (3)	−0.003 (2)	0.008 (3)
N5	0.037 (3)	0.035 (4)	0.048 (4)	−0.008 (3)	−0.009 (3)	0.019 (3)
N6	0.028 (3)	0.041 (4)	0.064 (5)	−0.004 (3)	−0.007 (3)	0.011 (3)
C10	0.035 (4)	0.033 (4)	0.024 (4)	−0.001 (4)	−0.006 (3)	−0.004 (3)
C11	0.023 (3)	0.038 (4)	0.027 (4)	−0.001 (3)	−0.003 (3)	−0.001 (4)
C12	0.031 (4)	0.025 (4)	0.035 (4)	−0.002 (3)	−0.001 (3)	−0.002 (3)
C13	0.029 (4)	0.040 (5)	0.027 (4)	0.006 (4)	−0.002 (3)	0.005 (4)
C14	0.022 (3)	0.050 (5)	0.043 (5)	0.000 (4)	−0.005 (3)	−0.001 (4)
C15	0.026 (3)	0.039 (4)	0.041 (4)	−0.006 (4)	0.001 (3)	0.012 (4)
C16	0.027 (4)	0.052 (5)	0.056 (6)	−0.008 (4)	0.000 (4)	0.005 (4)
C17	0.027 (4)	0.031 (4)	0.033 (4)	0.004 (3)	−0.007 (3)	0.004 (3)
C18	0.030 (4)	0.032 (4)	0.038 (4)	−0.004 (4)	−0.002 (3)	−0.005 (4)

### Geometric parameters (Å, º)

**Table:**

O1—C3	1.365 (8)	C1—C8	1.444 (9)
O1—C7	1.418 (7)	C2—C3	1.386 (9)
O2—C4	1.373 (8)	C3—C4	1.379 (9)
O3—C9	1.232 (8)	C4—C5	1.371 (9)
O2—H2A	0.8200	C5—C6	1.383 (8)
O4—C12	1.365 (8)	C2—H2	0.9300
O4—C16	1.426 (7)	C5—H5	0.9300
O5—C13	1.374 (8)	C6—H6	0.9300
O6—C18	1.244 (8)	C7—H7C	0.9600
O5—H5A	0.8200	C7—H7A	0.9600
N1—C8	1.259 (9)	C7—H7B	0.9600
N1—N2	1.377 (8)	C8—H8	0.9300
N2—C9	1.375 (7)	C10—C11	1.409 (9)
N3—C9	1.335 (9)	C10—C15	1.384 (8)
N2—H2B	0.8600	C10—C17	1.456 (8)
N3—H3B	0.8600	C11—C12	1.388 (9)
N3—H3A	0.8600	C12—C13	1.404 (9)
N4—N5	1.375 (7)	C13—C14	1.373 (9)
N4—C17	1.282 (7)	C14—C15	1.372 (10)
N5—C18	1.353 (9)	C11—H11	0.9300
N6—C18	1.336 (9)	C14—H14	0.9300
N5—H5B	0.8600	C15—H15	0.9300
N6—H6B	0.8600	C16—H16A	0.9600
N6—H6A	0.8600	C16—H16B	0.9600
C1—C2	1.386 (9)	C16—H16C	0.9600
C1—C6	1.390 (8)	C17—H17	0.9300
			
C3—O1—C7	117.9 (5)	C5—C6—H6	119.00
C4—O2—H2A	109.00	H7A—C7—H7C	109.00
C12—O4—C16	117.8 (5)	H7A—C7—H7B	109.00
C13—O5—H5A	109.00	O1—C7—H7B	109.00
N2—N1—C8	116.3 (5)	O1—C7—H7C	109.00
N1—N2—C9	121.6 (5)	O1—C7—H7A	109.00
C9—N2—H2B	119.00	H7B—C7—H7C	110.00
N1—N2—H2B	119.00	C1—C8—H8	119.00
C9—N3—H3A	120.00	N1—C8—H8	118.00
H3A—N3—H3B	120.00	C11—C10—C15	119.1 (5)
C9—N3—H3B	120.00	C15—C10—C17	119.2 (5)
N5—N4—C17	114.3 (5)	C11—C10—C17	121.6 (5)
N4—N5—C18	122.7 (5)	C10—C11—C12	119.4 (5)
C18—N5—H5B	119.00	C11—C12—C13	119.5 (6)
N4—N5—H5B	119.00	O4—C12—C11	126.7 (6)
H6A—N6—H6B	120.00	O4—C12—C13	113.7 (5)
C18—N6—H6A	120.00	O5—C13—C14	119.4 (5)
C18—N6—H6B	120.00	O5—C13—C12	119.6 (5)
C2—C1—C6	118.0 (6)	C12—C13—C14	121.0 (6)
C2—C1—C8	120.9 (6)	C13—C14—C15	119.0 (6)
C6—C1—C8	121.1 (6)	C10—C15—C14	121.9 (6)
C1—C2—C3	120.7 (6)	N4—C17—C10	122.8 (5)
O1—C3—C2	126.4 (5)	N5—C18—N6	115.7 (6)
C2—C3—C4	120.0 (6)	O6—C18—N5	120.4 (6)
O1—C3—C4	113.5 (5)	O6—C18—N6	123.8 (6)
O2—C4—C5	119.0 (6)	C10—C11—H11	120.00
O2—C4—C3	120.7 (6)	C12—C11—H11	120.00
C3—C4—C5	120.4 (6)	C13—C14—H14	120.00
C4—C5—C6	119.3 (5)	C15—C14—H14	120.00
C1—C6—C5	121.6 (6)	C10—C15—H15	119.00
N1—C8—C1	123.1 (6)	C14—C15—H15	119.00
O3—C9—N2	120.3 (6)	O4—C16—H16A	110.00
N2—C9—N3	115.6 (6)	O4—C16—H16B	109.00
O3—C9—N3	124.1 (5)	O4—C16—H16C	110.00
C3—C2—H2	120.00	H16A—C16—H16B	109.00
C1—C2—H2	120.00	H16A—C16—H16C	109.00
C6—C5—H5	120.00	H16B—C16—H16C	109.00
C4—C5—H5	120.00	N4—C17—H17	119.00
C1—C6—H6	119.00	C10—C17—H17	119.00
			
C7—O1—C3—C2	6.5 (9)	O1—C3—C4—O2	3.3 (8)
C7—O1—C3—C4	−176.1 (6)	O1—C3—C4—C5	−177.0 (5)
C16—O4—C12—C11	2.9 (9)	C2—C3—C4—C5	0.6 (9)
C16—O4—C12—C13	−175.3 (6)	C3—C4—C5—C6	0.4 (9)
C8—N1—N2—C9	172.7 (6)	O2—C4—C5—C6	−180.0 (5)
N2—N1—C8—C1	176.9 (5)	C4—C5—C6—C1	−1.5 (9)
N1—N2—C9—N3	−2.0 (8)	C15—C10—C11—C12	−0.8 (9)
N1—N2—C9—O3	179.3 (6)	C17—C10—C11—C12	175.9 (6)
C17—N4—N5—C18	−175.8 (6)	C11—C10—C15—C14	0.5 (9)
N5—N4—C17—C10	−176.6 (5)	C17—C10—C15—C14	−176.2 (6)
N4—N5—C18—O6	−177.8 (5)	C11—C10—C17—N4	1.8 (9)
N4—N5—C18—N6	0.3 (9)	C15—C10—C17—N4	178.5 (6)
C6—C1—C8—N1	175.4 (6)	C10—C11—C12—O4	−178.3 (6)
C2—C1—C6—C5	1.5 (9)	C10—C11—C12—C13	−0.2 (9)
C6—C1—C2—C3	−0.4 (10)	O4—C12—C13—O5	0.2 (8)
C8—C1—C2—C3	−178.8 (6)	O4—C12—C13—C14	179.8 (6)
C8—C1—C6—C5	179.8 (6)	C11—C12—C13—O5	−178.2 (5)
C2—C1—C8—N1	−6.3 (10)	C11—C12—C13—C14	1.4 (9)
C1—C2—C3—C4	−0.6 (10)	O5—C13—C14—C15	178.0 (6)
C1—C2—C3—O1	176.7 (6)	C12—C13—C14—C15	−1.7 (10)
C2—C3—C4—O2	−179.0 (6)	C13—C14—C15—C10	0.7 (10)

### Hydrogen-bond geometry (Å, º)

**Table:**

*D*—H···*A*	*D*—H	H···*A*	*D*···*A*	*D*—H···*A*
O2—H2*A*···O1	0.82	2.17	2.627 (6)	115
O2—H2*A*···O5^i^	0.82	2.34	3.108 (7)	156
N2—H2*B*···O6^ii^	0.86	2.11	2.923 (7)	158
N3—H3*A*···O6^iii^	0.86	2.16	2.987 (7)	162
N3—H3*B*···N1	0.86	2.31	2.674 (8)	106
O5—H5*A*···O4	0.82	2.18	2.632 (6)	115
N5—H5*B*···O3^iv^	0.86	2.08	2.909 (7)	161
N6—H6*A*···O3^v^	0.86	2.13	2.965 (7)	164
N6—H6*B*···N4	0.86	2.32	2.677 (7)	105

Symmetry codes: (i) −*x*, −*y*, *z*+1/2; (ii) *x*, *y*+1, *z*; (iii) *x*−1/2, −*y*, *z*; (iv) *x*, *y*−1, *z*; (v) *x*+1/2, −*y*+1, *z*.
